# New Policy of the Journal of Epidemiology Regarding the Relationship With the Tobacco Industry

**DOI:** 10.2188/jea.JE20170187

**Published:** 2018-01-05

**Authors:** Hiroyasu Iso, Keitaro Matsuo, Kota Katanoda, Takeo Fujiwara

**Affiliations:** 1 President, the Japan Epidemiological Association; Public Health, Osaka University Graduate School of Medicine, Osaka, Japan; 2 Editor-in-Chief, the Journal of Epidemiology; Division of Molecular and Clinical Epidemiology, Aichi Cancer Center Research Institute, Nagoya, Japan; 3 Deputy Editor, the Journal of Epidemiology; Center for Cancer Control and Information Services, National Cancer Center, Tokyo, Japan; 4 Deputy Editor, the Journal of Epidemiology; Department of Global Health Promotion, Tokyo Medical and Dental University, Tokyo, Japan

The Journal of Epidemiology, the official journal of the Japan Epidemiological Association, has decided not to consider for publication manuscripts on research carried out with funding from the tobacco industry and similar enterprises.^1^ This decision is based on awareness that the Japan Epidemiological Association has a responsibility to work to prevent tobacco-caused illnesses and mortality affecting the Japanese and world population, in view of the Japan Epidemiological Association’s aim of promoting advances in public health/healthcare and human well-being.

Tobacco is the greatest single cause of illness and death.^2^^,^^3^ An ever-accumulating body of scientific evidence supports this view, but the tobacco industry has attempted organized and strategic interventions to repudiate the findings, distorted facts, and committed scientific injustice.^4^^–^^8^ The tobacco industry has organized its own research and published results against the facts demonstrated by their data concerning the health impacts of secondhand smoke exposure,^9^^,^^10^ and they have interfered with the publication of research on the relationship of secondhand smoke exposure to lung cancer carried out by the International Agency for Research on Cancer.^6^^,^^11^ In the Cancer Prevention Study carried out in the United States by the American Cancer Society, the authors, who had an economic relationship with the tobacco industry, reported the absence of an association between secondhand smoke exposure and illness using a dataset which did not allow comparison between the exposure group and the non-exposure group. Notably, the report formally followed the rule of disclosing conflicts of interest (COI).^12^^,^^13^ However, in another analysis using an appropriate dataset from the Cancer Prevention Study, a significant association between secondhand smoke exposure and illness was detected.^14^ These lines of evidence indicate that interference, distortion of the facts, and injustice by the tobacco industry, acting against science, have been undertaken in a form that cannot readily be identified through disclosure of COI or the ordinary peer review process.^15^^,^^16^ These tactics of the tobacco industry remain unchanged to date, despite the health impacts of secondhand smoke exposure having been established scientifically.^17^

Secondhand smoke (ie, environmental tobacco smoke) was recognized as “carcinogenic in humans (Group 1)” in 2004 by the International Agency for Research on Cancer.^18^ Causal relationships of exposure to indoor secondhand smoke with lung cancer, ischemic heart disease, stroke, pediatric respiratory disease, sudden infant death syndrome, and other conditions have been scientifically established through comprehensive evaluations conducted by international agencies and governmental organizations.^19^^–^^21^ The tobacco industry in Japan (Japan Tobacco Inc.) has publicly asserted that the association between secondhand smoke exposure and illness has not been proven, without providing information to the public about the above-mentioned comprehensive scientific evaluations.^22^ Furthermore, the industry has been promoting organized campaigns against legislation to prevent indoor secondhand smoke exposure.^23^^,^^24^ Regarding a meta-analysis study demonstrating health hazards arising from secondhand smoke exposure in Japan,^25^ the Japanese tobacco industry cited two books on epidemiological methodology^26^^,^^27^ and claimed the evidence level of a meta-analysis to be lower than that of a single study.^28^ However, the two books cited at that time actually emphasize the importance of conducting meta-analyses in line with an appropriate method,^26^^,^^27^ so they do not support the claim made by the tobacco industry. Many other scientific errors contained in the industry’s claim have been clearly revealed and documented in the counterargument made by the National Cancer Center Japan.^29^

These facts illustrate that the tobacco industry has been manufacturing and distributing products serving as a major cause of illness and mortality and that it has been disseminating incorrect knowledge/views regarding health hazards from such products through its campaigns, which attempt to present a false veneer of scientific or academic activity. This is a point unique to the tobacco industry and can be viewed as a reason for the decision made by many medical journals to date. Many medical journals, including the British Medical Journal and its affiliates (Thorax, Heart, BMJ Open, Tobacco Control),^17^ the American Journal of Respiratory and Critical Care Medicine,^30^ PLoS Medicine and its affiliates (PLoS One and PLoS Biology),^31^ the British Journal of Cancer,^32^ Cancer Science (an official journal of the Japanese Cancer Association),^33^ and the Japanese Journal of Public Health^34^ have decided not to accept manuscripts on research financed by the tobacco industry, while admitting that this decision involves an aspect of social justice that would not be viewed as scientific.^17^^,^^35^ For a similar reason, the Japan Epidemiological Association has also decided not to consider for publication manuscripts on research carried out with funds obtained from the tobacco industry and similar enterprises.^1^ This decision has been made based on comprehensive assessment of the history of the relationship between epidemiology and the tobacco industry, the magnitude of tobacco’s health impacts, current social situations, and other relevant priorities. By making this decision, the Japan Epidemiological Association expresses its moral viewpoint that academic journals should not be utilized by the tobacco industry. The Japan Epidemiological Association will henceforth continue evaluating its relationship with the society as a whole, including the tobacco industry, corresponding to changes in social situations surrounding the implementation of epidemiology as a science.
